# Successful Trial of Octreotide and Ketorolac for the Management of Increased Biliary Drain Output: A Case Report

**DOI:** 10.7759/cureus.1421

**Published:** 2017-07-03

**Authors:** Fasil Tiruneh, Ahmad Awan, Abdullahi Musa, Daniel Chen

**Affiliations:** 1 Department of Internal Medicine, Howard University Hospital; 2 Internal Medicine, Washington Dc Va Medical Center

**Keywords:** percutaneous biliary drains, octreotide, ketorolac, increased biliary drainage

## Abstract

We describe a 69-year-old male patient with the status of obstructive jaundice post percutaneous biliary drainage for prior obstructive jaundice and who presented with a complaint of generalized weakness and increased output from the drainage tube. The patient developed worsening jaundice, which was noted to be obstructive in nature with a marked dilatation of the biliary tree and a distal obstruction of the common bile duct. Subsequently, a percutaneous biliary drain was placed for symptomatic management. However, the patient continued to have increased output from the drain, approximating 3-4 liters a day, which made the patient dependent on continuous intravenous hydration. The case presented a therapeutic challenge in reducing the drainage amount. We have tried a successful approach based on the physiologic effect of octreotide and nonsteroidal anti-inflammatory drugs (NSAIDs) in the formation of bile secretion. This approach has not been clearly described in the literature. We highlight the importance of further study to validate the use of these medications in similar clinical scenarios.

## Introduction

Biliary obstruction can be caused by gallstones, scarring from injury, or cancer. This prevents bile from being transported to the intestine. However, the amount of biliary drainage varies depending on several factors, including hormonal variations and the cause of the obstruction. Patients with malignant obstructions are usually managed symptomatically with biliary drainage tube or catheter placement, which can be used to relieve a blockage in the bile duct, either permanently or as a temporary solution, before definite treatment such as surgery. The drain can be placed percutaneously through the liver and to enable percutaneous transhepatic biliary drainage (PTBD) or can be performed as part of percutaneous transhepatic cholangiography by interventional radiology.

Some common complications after biliary decompression are cholangitis and catheter-related problems. In a study including 73 patients who underwent biliary decompression using PTBD, 7 patients had episodes of cholangitis and 8 patients had catheter problems requiring either hospital admission or emergency room visits [1]. However, increased biliary output is rarely observed and creates a challenge in successfully suppressing the overproduction of bile.

## Case presentation

**Figure 1:**
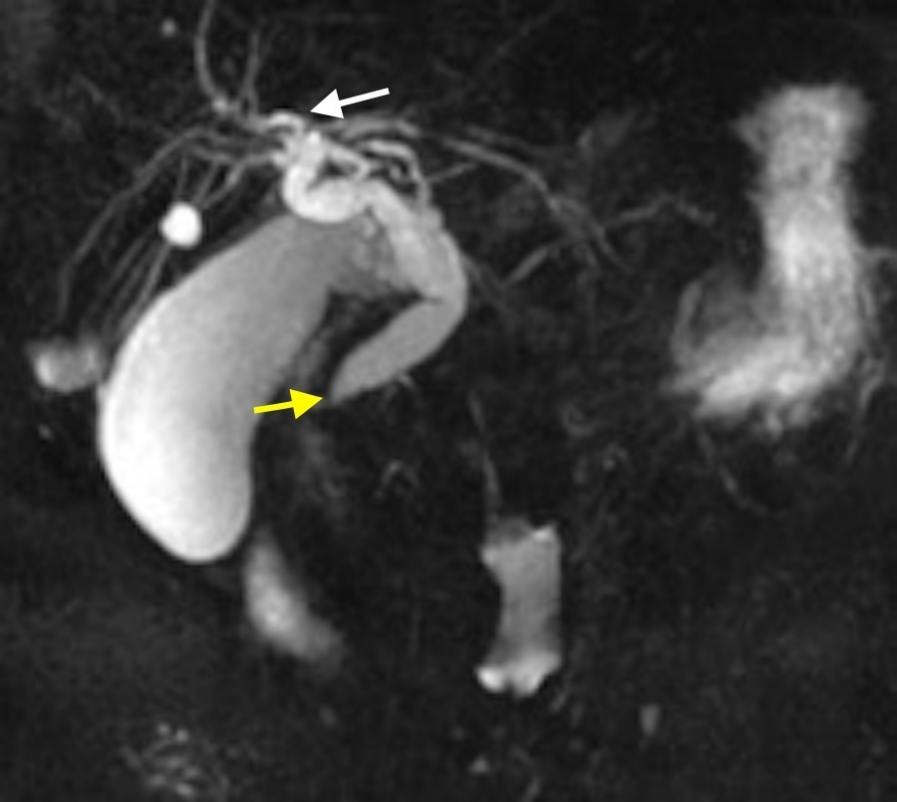
MRI showing dilated biliary ducts (white arrow) with tapering of the distal common bile duct (yellow arrow)

**Figure 2:**
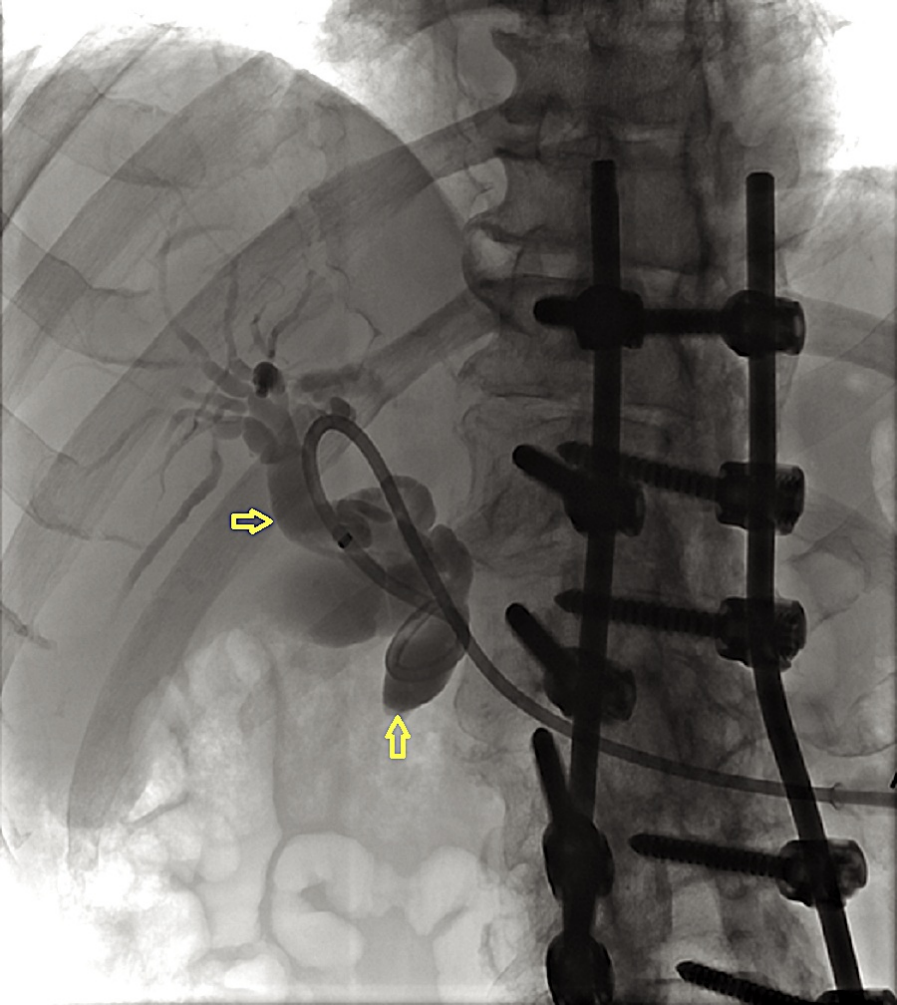
Retrograde cholangiopancreatography showing a tortuously dilated common bile duct and intrahepatic biliary ducts (yellow arrows) with a biliary stent in place

**Figure 3:**
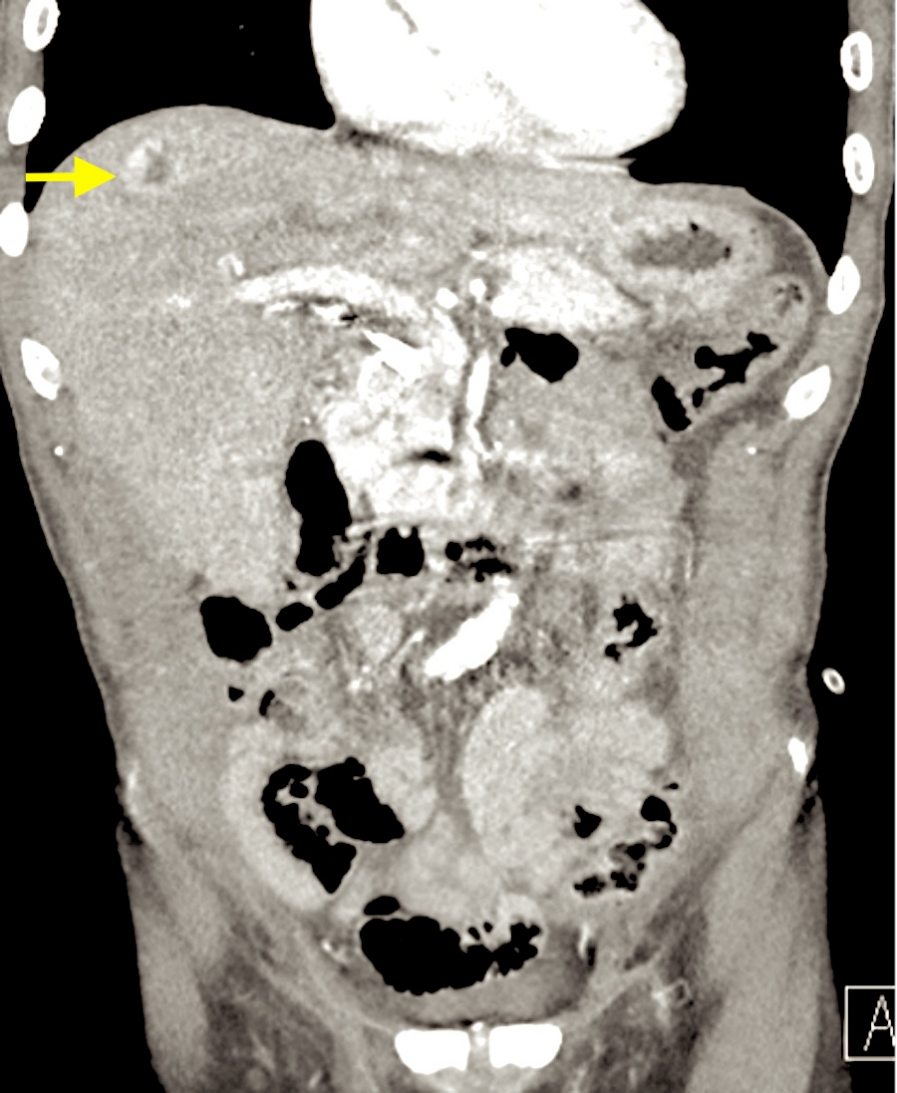
CT scan showing intensely enhancing mass in segment 8 of the liver (yellow arrow)

A 69-year-old African American male with a past medical history of hepatitis C virus, genotype-1b, and infection treated with ombitasvir/paritaprevir/ritonavir with sustained virologic response, presented to the hospital with anorexia, nausea, vomiting, and generalized weakness. The patient was hospitalized for obstructive jaundice two months prior to his current presentation. Magnetic resonance cholangiopancreaticography during his prior admission showed moderate intra and extrahepatic biliary duct dilatation with abrupt tapering of the distal common bile duct. For this, an attempt to cannulize the ampulla using endoscopic retrograde cholangiopancreaticography (ERCP) was unsuccessful because of redundant periampullary tissue folds. Subsequently, a percutaneous biliary drain was placed by interventional radiology for symptomatic improvement. The patient had since then increased output from the drain to around 3-4 liters. Additional imaging studies using ultrasound revealed nonspecific hepatic hypoechoic nodules. These findings were further characterized by computed tomography (CT), which demonstrated several enhancing liver lesions which were highly suspicious for metastatic disease (Figures 1-3).

On his current admission, the patient presented with blood pressure: 98/60 mmHg, pulse rate: 100 beats per minute, respiratory rate: 16 breaths per minute, and temperature: 97^o^F. On physical examination, the patient appeared hypovolemic. Laboratory studies were notable for acute kidney injury and conjugated hyperbilirubinemia with creatinine: 5.8 mg/dL, total bilirubin: 9.8 mg/dL, and direct bilirubin: 6.8 mg/dL. The patient was resuscitated with intravenous fluid with successful resolution of acute kidney injury.

The internalization of the drain was attempted by interventional radiology two times; however, both attempts were unsuccessful because of difficult anatomy and technical difficulties. Biliary drain output increased to more than 4 L/day after the procedure. On hospital stay Day 4, capping of the drain was attempted to cut down fluid losses. However, on Day 5, the patient developed a fever of 101.2^o^F. Blood cultures were positive for Brevundimonas vesicularis, which was found to be sensitive to fluoroquinolones. The patient eventually completed two weeks of antibiotic therapy with ciprofloxacin and metronidazole with an improvement of his sepsis.

**Figure 4:**
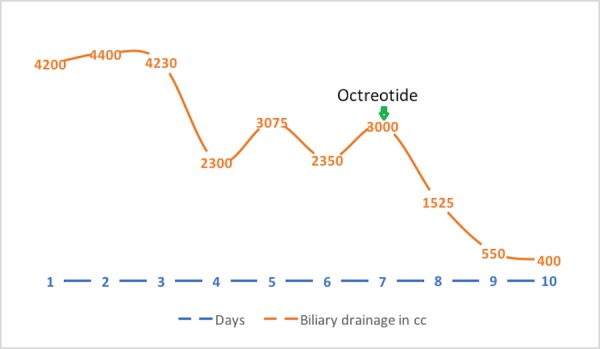
Graph showing daily biliary drainage in cubic centimeter (cc) Green arrow shows when octreotide was started

**Figure 5:**
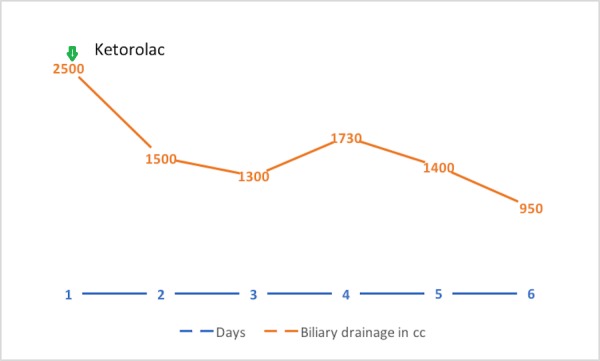
Graph showing daily biliary drainage in cubic centimeter (cc) Green arrow shows the start date for ketorolac

Despite the treatment of the infection, the patient continued to have persistently high biliary drain output and remained dependent upon intravenous fluid to replace losses. That led us to try unconventional measures. We initiated a trial of octreotide 100 mcg q8 sc tid. After receiving three doses, the drain output decreased to 550 cc (Figure 4). The effect persisted for a couple of more days. However, due to convenience and the availability of short-acting octreotide, the patient was discharged on long-acting depot octreotide 20 mg intramuscular. However, the patient presented again with dehydration and acute kidney injury, which resolved with fluid resuscitation. At this point, we attempted a trial of intravenous ketorolac to improve the drain output. This resulted in a moderate improvement of the drain from 2500 cc to 1500 cc on Day 2 and, subsequently, to 950 cc on Day 6 (Figure 5). Eventually, a liver biopsy was performed, which showed hepatocellular carcinoma. The patient was referred to a tertiary care center for further management.

## Discussion

Percutaneous biliary drains are routinely placed in patients to relieve obstructions [2]. Persistent high output is a rare complication of a percutaneous biliary drain. Increased output is usually managed by the internalization of the drain, biliary stenting, or surgery. However, when patients are either poor candidates for procedural interventions or when interventions are unsuccessful, as in our case, therapeutic options are limited to support with intravenous fluids, which is not possible outside the hospital setting. At this point in time, little, if any, literature exists to provide guidance on the medical management of sustained high-output biliary drainage when definitive procedures fail.

Secretin receptors are exclusively expressed at the basolateral membrane of cholangiocytes; when they are stimulated, the intracellular levels of cyclic adenosine monophosphate (cAMP) are increased [3]. This leads to the release of bile. Dopamine, somatostain, and, to some extent, gastrin inhibit basal- and secretin-based bicarbonate-rich bile [4-5]. On the other hand, vasoactive intestinal peptide (VIP) increases secretin-stimulated bile flow. In one study involving 12 patients with complete biliary fistulas, VIP infusion increased bile volume by 60%, and combined VIP and secretin infusion increased bile volume by another 70% [6].

Octreotide is commonly used to manage diarrhea in the carcinoid syndrome. Typically, patients should be stabilized for at least two weeks before switching to long-acting depot [7]. In our case, we used short-acting octreotide with good benefit. Octreotide exerts its effect by inhibiting serotonin release and the secretion of gastrin, VIP, insulin, glucagon, secretin, motilin, and pancreatic polypeptide and inhibiting pancreatic exocrine secretion [8]. This theoretically leads to a decrease in bile secretion. To the best of our knowledge, octreotide has never been used before for the management of persistent high output from the biliary drain. Potential side effects are either local (pain, redness, or swelling) or gastrointestinal (flushing, constipation, gallstones, and cholecystitis). Another important consideration is the cost of octreotide and the logistics of administering injections.

Non-steroidal anti-inflammatory drugs (NSAIDs) help improve biliary colic and have an anti-inflammatory effect. Prostaglandins (PGs) are important mediators in the contraction of the biliary tract; blocking prostaglandins will result in an inhibition of the contraction of the bile duct and will potentially result in an improvement in drain output [9]. In our patient, the use of ketorolac resulted in mild to moderate improvement in bile drain output; however, its beneficial effect on the biliary drain was not as potent as was observed with the use of octreotide. The major limitations of ketorolac include gastrointestinal bleed and kidney injury; therefore, it is typically recommended only for short-term use.

## Conclusions

Persistant high output from the biliary drain is a rare but serious complication of percutaneous biliary drainage, especially in patients with associated malignancies. Increased output is usually managed surgically or by the internalization of the drain. Therapeutic options for patients who are not surgical candidates are currently lacking beyond supportive care with intravenous fluids. The capping of the drain after a specific amount is also seen in common practice; however, the risk of infection increases because of that. Octreotide helps in reducing the amount of output from the drain; however, the cost and approval from insurance companies remain a major problem for the effective use of the medication. In selected patients with normal kidney function, we can use NSAIDs. However, further research and double-blinded studies are required to fully assess the magnitude of the benefits and side effects.
